# The Relationship of Phone Calls and Social Network Service Contact with Depression

**Published:** 2019-07

**Authors:** Eunmi LEE, Yujeong KIM

**Affiliations:** 1. Department of Nursing, Hoseo University, Asan, Republic of Korea; 2. College of Nursing, Kyungpook National University, Daegu, Republic of Korea

## Dear Editor-in-Chief

The average prevalence rate of depression in university students was 30.6%, while the same rate among Korean university students was 38% (1). Recently, changes in interpersonal relationships as a result of the use of social network services (SNS) has been identified as a primary factor affecting depression in university students. It has been reported that 72.7% of university students in Korea connect to SNS everyday (2). SNS may be used to create a false persona rather than to reveal one’s true self. Individuals may compare themselves with others on SNS in terms of happiness, attractiveness, accomplishments, and diversity of relationships, evaluate themselves poorly, and belittle themselves. In such cases, individuals may experience depression due to a relative sense of inferiority and deprivation (3). Research has primarily been carried out on SNS usage time and connection frequency; however, few studies have examined the relation of the number of people contacted via SNS, with depression (4). Meanwhile, in comparison to SNS, contact and communication through phone calls has been shown to have a maintenance and exchange effect on social relationships, similar to face-to-face communication, and that more intimate emotional and personal information exchanges are possible through phone calls as opposed to face-to-face communication (5). However, there is a lack of studies on the relation of the number of people contacted through voice communications, with depression among university students.

This study aimed to identify the relationship of phone calls and social network service (SNS) contact with depression among Korean university students. This study employed a survey design to identify the relation of the number of people contacted via phone calls and SNS with depression. Data were collected from 460 undergraduates regarding the number of people they contacted via phone and SNS, and their scores on the Centres for Epidemiological Studies-Depression scale (CES-D). For all people contacted (family and non-family), depression decreased with higher numbers of people contacted via phone calls (β=−0.144, *P*=.028), and increased when higher numbers of people were contacted via SNS (Model 1: β=0.119, *P*=.017, Model 2: β=0.122, *P*=.022). Further, depression decreased with higher numbers of meaningful contacts (both family and non-family), and there were statistically significant differences in both Model 1 and Model 2 (Model 1: β=−0.153, *P*=.002, Model 2: β=−0.152, *P*=.002).

We found that depression decreased in university students when higher numbers of people were contacted via phone calls. As an intervention method for university students with a propensity for depression, a strategy is needed for strengthening social support and for reducing the negative effects of depression through regular phone calls with family and friends through which emotions can be exchanged. When students contact higher numbers of people via SNS, it may cause them to feel disappointed in themselves and inferior through upward social comparisons, and depression may increase due to a diminishing sense of happiness (3). To reduce depression among university students owing to the SNS images of others, there is a need to strengthen various university programmes that help students recognise that the happy images of others are not their entire lives.
